# Preconditioning with levosimendan reduces postoperative low cardiac output in moderate-severe systolic dysfunction patients who will undergo elective coronary artery bypass graft surgery: a cost-effective strategy

**DOI:** 10.1186/s13019-020-01140-z

**Published:** 2020-05-24

**Authors:** Juan José Jiménez-Rivera, Andrea Álvarez-Castillo, Jorge Ferrer-Rodríguez, José Luis Iribarren-Sarrías, Martín Jesús García-González, Pablo Jorge-Pérez, Juan Lacalzada-Almeida, Rosalía Pérez-Hernández, Javier Montoto-López, Rafael Martínez-Sanz

**Affiliations:** 1 Department of Intensive Care, Hospital Universitario de Canarias, La Laguna Tenerife, S.C.Tenerife, Canary Islands Spain; 2 Health Economist, University of La Laguna, La Laguna, Tenerife, S.C.Tenerife, Canary Islands Spain; 3 Department of Cardiology, Hospital Universitario de Canarias, La Laguna, Tenerife, S.C.Tenerife, Canary Islands Spain; 4 Department of Cardiovascular Surgery, Hospital Universitario de Canarias, La Laguna, Tenerife, S.C.Tenerife, Canary Islands Spain

**Keywords:** Cost-effectiveness, Preconditioning, Levosimendan, Cardiac surgery, Low cardiac output, Coronary artery bypass graft

## Abstract

**Background:**

Patients with moderate-severe systolic dysfunction undergoing coronary artery bypass graft have a higher incidence of postoperative low cardiac output. Preconditioning with levosimendan may be a useful strategy to prevent this complication. In this context, design cost-effective strategies like preconditioning with levosimendan may become necessary.

**Methods:**

In a sequential assignment of patients with Left Ventricle Ejection Fraction less than 40%, two strategies were compared in terms of cost-effectiveness: standard care (*n* = 41) versus preconditioning with Levosimendan (*n* = 13). The adverse effects studied included: postoperative new-onset atrial fibrillation, low cardiac output, renal failure and prolonged mechanical ventilation. The costs were evaluated using deterministic and probabilistic sensitivity analysis, and Monte Carlo simulations were performed.

**Results:**

Preconditioning with levosimendan in moderate to severe systolic dysfunction (Left Ventricle Ejection Fraction < 40%), was associated with a lower incidence of postoperative low cardiac output in elective coronary artery bypass graft surgery 2(15.4%) vs 25(61%) (*P* < 0.01) and lesser intensive care unit length of stay 2(1–4) vs 4(3–6) days (*P* = 0.03). Average cost on levosimendan group was 14,792€ while the average cost per patient without levosimendan was 17,007€. Patients with no complications represented 53.8% of the total in the levosimendan arm, as compared to 31.7% in the non-levosimendan arm. In all Montecarlo simulations for sensitivity analysis, use of levosimendan was less expensive and more effective.

**Conclusions:**

Preconditioning with levosimendan, is a cost-effective strategy preventing postoperative low cardiac output in patients with moderate-severe left ventricular systolic dysfunction undergoing elective coronary artery bypass graft surgery.

## Background

Revascularization surgery plays an important role in patients with left ventricular dysfunction and major vessel involvement [1]. The benefit of such revascularization on survival or hospitalization for cardiovascular cause by these patients is affected by the increased risk of mortality in the first 30 days postintervention [2]. The minimization of this risk through the use of levosimendan has been studied in three large randomized trials recently conducted: LICORN [3], LEVO-CTS [4] and CHEETA [5]. None of them showed a clear decrease in mortality in the levosimendan group, although in a post hoc analysis of LEVO-CTS [4] study was found that in patients operated exclusively on Coronary Artery Bypass Grafting (CABG) was beneficial. Although the definitions of postoperative low cardiac output (LCO) are variable among the studies cited, only LEVO-CTS [4] study was found beneficial against the reduction of postoperative LCO.

Economic evaluations of this drug are largely focused in the setting of heart failure [6, 7], with little evidence in the field of cardiac surgery. A recent study evaluated the cost-benefit of levosimendan compared to dobutamine in the perioperative of cardiac surgery in a national care register. In this study, medication and postoperative complications were the clinical variables analysed, and on the cost-benefit side, costs and days of stay were evaluated [8]. However, it has not been evaluated whether preconditioning with this drug is cost-effective. The double purpose of this study was to asses the effectiveness of preconditioning with levosimendan in order to decrease postoperative LCO in patients with impaired left ventricle ejection fraction (LVEF) lower than 40% [3], who will undergo elective isolated CABG surgery compared to optimal standard care without levosimendan and to carry out an economic evaluation comparing both strategies, following ISPOR CHEERS methodology.

## Methods

The study was approved by the local clinical Research Ethics Committee of the center. It was conducted in accordance with the Declaration of Helsinki concerning medical research in human subjects. All prospective patients signed an informed consent form. (See “patients information sheet” and “informed consent form for patients” as supplemental material). Regards to historical cohort, Clinical Research Ethics Committee allowed this study to use their clinical-healthcare data without informed consent.

### Rationale for study

Impaired LVEF is a well-known risk factor for postoperative complications as LCO syndrome [2]. In the context of a Quality Management System for the cardiac surgical patient of our center we detected an increased incidence of postoperative low cardiac output syndrome in patients with moderate-severe systolic dysfunction (retrospective cohort). This was perceived as a bottleneck limiting downstream the adequate flow of patients from the cardiac surgery program. For this reason, we implemented an improvement action, consisting of preconditioning with levosimendan to these patients in a sequential way (prospective cohort). In order to assess the effectiveness of this measure and perform an additional economic analysis we had to resort to an ambispective design.

The ambispective cohorts study consisted in two parts:

#### Part 1. Assessment of preconditioning with levosimendan on the postoperative complications in high risk-patients

##### Study population

Patients were eligible if they fulfilled the following criteria: 1) elective isolated CABG, and 2) impaired LVEF lower than 40%. Exclusion criteria were severe liver disease, chronic obstructive pulmonary disease (COPD) and redo patients.

### Historical cohort

We performed a retrospective review of our institutional prospectively maintained database in adult patients undergoing cardiac surgery from January 2010 to December 2015. Selected patients fulfilled inclusion criteria for analysis were admitted 24 h before intervention to the general cardiovascular surgery ward.

### Prospective cohort

From January 2016 to January 2018 following a sequential assignment all patients fulfilled eligible criteria were admitted 48 h before the intervention for preconditioning with levosimendan. The dosage of Levosimendan, from 0.05 to 0.2 mcg/Kg/min for 24 h, selected for this study was based on previous studies and clinical research experiences [9]. It is also similar to that used in previous studies in the same clinical context [4, 10, 11]. The loading dose was omitted for safety reasons and the infusion dose could be reduced to 0.05 μg/kg/min or stopped if the patient’s response was considered excessive to its administration (hypotension or tachycardia). Suspension of administration for this reason was considered a serious adverse event. However, no adverse events were observed during levosimendan infusion. Patients were monitored in a Coronary Unit. Once the infusion was over, the patients were transferred to a general cardiovascular surgery ward for another 24 h after which the surgery was performed (48 h after the beginning of levosimendan infusion).

### Data collection

Demographic variables, comorbid conditions, perioperative clinical data, postoperative outcomes (hemodynamics, need for amines, disturbance of cardiac rhythm, duration of mechanical ventilation, postsurgical ICU and hospital stay and mortality) were recorded. Biochemical determinations at ICU admission, at 4 h and 24 h after surgery as well as maximum serum levels of biomarkers during ICU stay, were recorded.

The main postoperative complications studied were: A) postoperative new-onset atrial fibrillation (episodes of non-self-limited atrial fibrillation with at least 30 s duration in the postoperative period) [12], B) low cardiac output (defined as an haemodynamic picture with a measured cardiac index lower than 2.2 l/min/m^2^, without associated relative hypovolemia, or compatible clinical picture presenting oliguria, central venous saturation < 60% and/or lactate > 3 mmol/L; or cardiogenic shock: cardiac index < 2.0 l/min/m2, with systemic blood pressure < 90 mmHg, without relative hypovolemia and with oliguria) [13], C) renal failure (increase 1.5 times of the preoperative creatinine value) [14] and D) prolonged mechanical ventilation (longer than 24 h) [15].

### Perioperative management

Anesthetic procedures were standardized and consisted of an opioid-based anesthetic supplemented with volatile anesthetic and muscle relaxants. All interventions were performed by the same surgical team with wide experience in these surgical interventions. All patients were preoperatively monitored with a pulmonary artery continuous thermodilution catheter (Edwards Lifesciences LLC, Irvine, CA, USA), calibrated at least twice a day following our internal protocol unit. Neither heparin coated circuits nor leukocyte filters were used. The extracorporeal circuit consisted of a hardshell membrane oxygenator (Optima XP; Cobe, Denver, CO, USA, or Quantum Lifestream International, Inc., Woodlands, TX, USA), a Tygon™ (Dideco s.r.l., Mirandola, Italy) extracorporeal circuit, and a Medtronic™ Biopump (Medtronic, Inc., Minneapolis, MN, USA) centrifugal pump. Temperatures were maintained around 32 °C to 34 °C, the pump flow was adjusted to maintain a mean arterial pressure of greater than 60 mmHg and a flow index of 2.2 L/minute per square meter. Myocardial protection was achieved using antegrade, cold, St. Thomas 4:1 sanguineous cardioplegia. Fluid management was carried out to achieve 8 to 12 mmHg of central venous pressure or 12 to 15 mmHg of pulmonary artery occlusion pressure at zero positive end-expiratory pressure by infusions of crystalloids. Catecholamine support, when necessary, was used as follows: Norepinephrine was titrated to achieve a mean arterial pressure of greater or equal to 70 mmHg, and dobutamine was titrated to achieve a cardiac index of greater or equal to 2.5 L/minute per square meter. Amines were tapered off in steps of 0.02 and 1 μg/kg per minute, respectively. Patients were extubated when he presented hemodynamic stability, a Glasgow Coma Score > 13, decreased chest tube bleeding, good oxygenation and ventilation and adequate muscular strength and cough capacity. For more details of hemodynamic and weaning protocols see supplemental material (Additional files 1 and 2).

### Statistical analysis

Comparison between cohorts (high-risk patients with and without preconditioning with levosimendan) were conducted by applying Pearson’s χ2 test or Fischer’s exact test for categorical variables, and the Student’s t-test or the Mann-Whitney’s U test for continuous variables, as appropriate. Mantel-Haenszel test was used to assess levosimendan effect on Left Ventricular Ejection Fraction (LVEF) strata groups on the prevention of postoperative LCO syndrome. Statistical significance was defined as a *P*-value of less than 0.05. SPSS statistics v20 (IBM SPSS Statistics) was used for clinical analysis.

#### Part 2. Economic evaluation comparing preconditioning with levosimendan vs optimal standard care

The two strategies referred to above were compared in terms of costs and health results in the second part of this study, in the context of the hospital setting.

##### Overview model

A decission tree was created to represent the intervention and its health outcomes during the post-intervention process for a time period ranging from intervention to hospital discharge. Thereby, this analytical model allows us to represent the potential set of outcomes for both patient cohorts.

The analysis uses the perspective of the National Health Service, i.e. taking into account the health costs incurred by this service. The benefits for the patient are measured in postoperative complications avoided. No discount has been applied to the costs of the results due to the time horizon considered.

The evaluation is based on a decission model that synthesizes information obtained through clinical records and internal accounting of the associated costs of the cardiac surgical patient process and the effectiveness of the program.

From the first and main decission node of both trees, two branches start, one where the patient is administered levosimendan and the other one without its administration. From these two initial branches, LCO, a potential complication after cardiac surgery, may or may not occur in relation to the previous development of atrial fibrillation and increases the incidence of renal failure and prolonged mechanical ventilation and, therefore, longer length of Intensive Care Unit (ICU) and hospital stay (LOS), and associated costs. Renal failure and prolonged mechanical ventilation may or may not be related to LCO.

##### Model inputs

To construct the model three types of data can be grouped: probabilities of transition between states for the cardiac-surgical process use of hospital resources and unit costs of the resources used.

##### Effectiveness

These two strategies were developed in elective CABG patients with an ejection fraction less than 40%. The historical cohort of patients was admitted to the hospital ward the day before the intervention. Subjects of levosimendan group were admitted 48 h before surgery as explained before. Immediate postoperative required ICU admission. On the other hand, the incorporation of antiarrhythmic prophylaxis with amiodarone was ruled out, since most of the patients included in the present study start from a basic antiarrhythmic treatment with beta-blockers due to their condition of ischemic heart disease.

The adverse events studied were: A) postoperative new-onset atrial fibrillation, B) low cardiac output, C) renal failure and D) prolonged mechanical ventilation. The monitoring care units (Coronary and ICU) and hospital LOS is also taken into account.

##### Resources

After surgery, a specific blood test is performed, which includes haemogram, general biochemistry and coagulation, together with a daily electrocardiogram and blood gases during the stay in the ICU. The usual postoperative medication of the CABG surgery patient was included. In addition, at least one echocardiogram is performed during ICU stay, and an electrocardiogram is performed in the general ward before discharge (Table 1).

**Table 1:** Costs taken from hospital accounting

UNITS	COSTS (€)
***PROCEDURES***	
Surgery	6269.20
Hemofilter kit	173.82
Mechanical ventilation at ICU	460.00
***SUPPLEMENTAL TESTS***	
Laboratory profile*	85.96
Electrocardiogram	20.37
Echocardiogram	120.36
***MEDICATION***	
Amiodarone prophylaxis (tablet)	0.11
Amiodarone 150 mg (IV ampoule)	0.25
Furosemide 20 mg (IV ampoule)	0.13
Atorvastatin 20 mg (tablet)	0.07
Bisoprolol 5 mg (tablet)	0.08
Acetylsalicylic acid 100 mg (tablet)	0.03
Dalteparin 5000 IU (SC, prefilled syringe)	0.31
Acetaminophen 1 g IV (vial)	0.53
Norepinephrine 10 mg (1 vial)	0.95
Dobutamine 250 mg (1 vial)	1.38
Levosimendan	581.63
Oral paste/day	5.50
Decontaminating solution	10.00
Effluent bag	8.98
***STAYS***	
Coronary care unit	909.79
ICU	1266.95
Hospital ward	234.12

€: euro, ICU: intensive care unit, IV: intravenous

##### Costs of adverse events

The costs of adverse events are mainly those arising from a longer stay and the specific resources needed for each complication. In case of A)s atrial fibrillation, antiarrhythmic medication is required, and in some cases cardioversion must be conducted. In presence of B) LCO, an echocardiogram is performed daily in addition to the administration of vasoactive amines and inotropic agents. When C) renal failure occurs, replacement therapy is occasionally implemented; and for D) mechanical ventilation, selective digestive decontamination therapy is given as ICU protocol. For all the situations mentioned above the increase in LOS is implicit.

##### Deterministic sensitivity analysis

In order to know the robustness of the model results at the prospect possible uncertainty in the parameter values, and to facilitate their comparison with the results obtained from other publications, a univariate deterministic sensitivity analysis was carried out. In this analysis, the percentage of LCO was varied according to the data published in studies of similar nature [4, 16, 17]. This parameter was selected due to the variability of the definitions used in the post-operative context of cardiac surgery and due to the impact that its appearance entails in the morbidity of these patients.

##### Probabilistic analysis

To characterize the uncertainty in the model, we performed a probabilistic sensitivity analysis using Monte Carlo simulation, applying probability distributions to each parameter according to its homoscedasticity.

Transition probabilities are generally characterized by a beta distribution that is defined by the parameters that represent the occurrence and non-occurrence of an event.

Resource use was characterized using a uniform distribution, while gamma distributions were applied to cost parameters; in this case, we used an upper and lower limit of 10% around the mean values.

We ran 10,000 simulations on the Monte Carlo analysis. For each simulation, we obtained the average cost and the number of patients free of adverse events that we represented in the cost-effectiveness plane.

## Results

### Part 1: clinical results

**Fig. 1:**
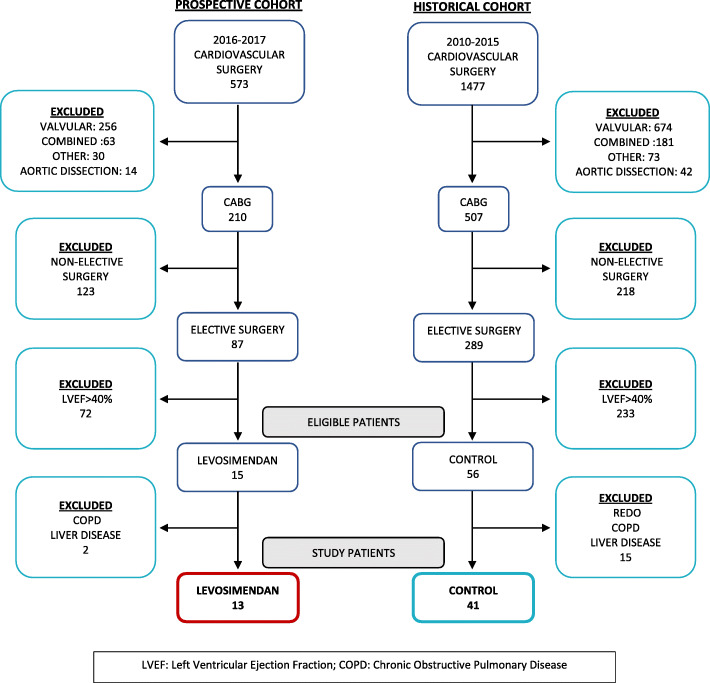
Flow chart patients

We analysed 41 out of 1477 patients from historical cohort and 13 out of 573 patients from prospective cohort, who fulfilled the inclusion and had no exclusion criteria, (Fig. 1).

Mean LVEF was 30% in levosimendan group versus 39% in the control group, (*P* < 0.01). From the 13 patients who received the drug before surgery, 5 of them presented LVEF between 35 and 40% and the other 8 patients had LVEF < 35%. Postoperative LCO was observed in 2 (15.3%) of patients in the levosimendan group compared to 25 (61%) in the control group (*P* < 0.01). See Table 2. When a stratified analysis was performed based on the LVFE the statistical significance was maintained. (*P* = 0.02). See Table 3. None of the patients in the levosimendan group presented cardiogenic shock versus 10 of them in the control group, (*P* = 0.047). Levosimendan group showed lower troponine I level and significantly lower lactic acid level at 24 h after surgery, (P = 0.02). Also, levosimendan group need less ventilation support (*P* < 0.01), lower incidence of new-onset atrial fibrillation, lower postoperative renal failure and reported a significantly shorter ICU stay than control group, (*P* = 0.03). Table 2.

**Table 2:** Patients characteristics in levosimendan and control groups

	LEVOSIMENDAN (n = 13)	CONTROL (*n* = 41)	*P*
*MEDICAL DEMOGRAPHICS*			
Age (years)*	68.6 ± 9.8	65.7 ± 12.2	0.43
Male, n (%)	12 (92.3)	29 (70.7)	0.11
Female, n (%)	1 (7.7)	12 (29.3)	
Arterial hypertension, n (%)	9 (69.2)	25 (61)	0.59
Dyslipidemia, n (%)	6 (46.2)	25 (61)	0.35
Diabetes mellitus, n (%)	7 (53.8)	19 (46.3)	0.35
Renal failure, n (%)	2 (15.4)	4 (9.8)	0.57
Previous myocardial infarction, n(%)	4 (36.4)	11 (32.4)	0.81
HLV	3 (23.1)	8 (19.5)	0.27
NYHA, n(%)I	3 (23.1)	16 (39)	0.78
II	4 (30.8)	10 (24.4)	
III	4 (30.8)	10 (24.4)	
IV	2 (15.4)	5 (12.2)	
Euroscore †	7.7 ± 6.9	10.4 ± 8.8	0.33
LVEF (fraction) †	30 (28–37)	39 (35–40)	< 0.01
*INTRAOPERATIVE*			
Graft type n(%) Saphenous	2 (15.4)	6 (14.6)	0.79
IMA	2 (15.4)	10 (24.4)	
Both	9 (69.2)	25 (61)	
Aortic cross clamp time (min)†	62 ± 18	62 ± 44	0.98
Cardiopulmonary bypass time (min)*	104 ± 35	96 ± 59	0.61
Pump reentry, n(%)	0	2 (4.9)	0.42
*MEDICAL-POSTOPERATIVE*			
Postoperative NOAF, n (%)	2 (15.3)	9 (21.9)	0.46
Low cardiac output, n (%)	2 (15.4)	25 (61)	< 0.01
Cardiogenic shock, n(%)	0	10 (24.4)	< 0.05
Posoperative myocardial infarction, n(%)	0	1 (6.2)	0.38
Troponine I at 24 h, ng/mL	3.7 ± 6.7	7.5 ± 10.9	0.26
Troponine I máximum, ng/mL	4.7 ± 5.8	16.9 ± 25.2	0,20
Lactic acid at 24 h, mmol/L	1.1 ± 0.4	4.1 ± 7.2	0.02
Hours of Dobutamine †	2 (0–24)	23 (3–71)	0.04
Hours of Norepinephrine †	0 (0–17)	0 (0–42)	0.33
Prolonged mechanical ventilation, n(%)	1 (7.7)	9 (22)	0.25
Hours of mechanical ventilation†	2 (2–5)	7 (5–20)	< 0.01
Renal failure, n (%)	1 (7.7)	10 (24.4)	0.19
Hemodialysis, n (%)	0 (0)	3 (7.3)	0.32
Exitus, n (%)	0 (0)	5 (12.2)	0.18
ICU LOS (days) †	2 (1–4)	4 (3–6)	0.03
PO Ward stay (days) †	6 (4–8)	5 (4–9)	0.78

The results are expressed as cases and percentages -n (%) - either as *arithmetic mean ± standard deviation, or † median and 25–75 percentile, NOAF: new-onset atrial fibrillation, Prolonged mechanical ventilation: > 24 h. LVEF: left ventricle ejection fraction. HLV: hypertrophy of left ventricle. NYHA: New York Heart Association. IMA: internal mammary artery. ICU LOS: Intensive Care Unit Length of stay; PO: Posoperative

**Table 3:** Low cardiac output in levosimendan and control groups according to LVEF

	LEVOSIMENDAN	CONTROL	*P*
LVEF 35–40%	(*n* = 5)	(*n* = 33)	0.01
Low cardiac output, n (%)	0	20 (60.6)	
LVEF < 35%	(*n* = 8)	(n = 8)	
Low cardiac output, n (%)	2 (25)	5 (62.5)	

The results are expressed as cases and percentages -n (%). LVEF: left ventricle ejection fraction. P expressed Mantel-Haenszel statistics

### Part 2: base case results

Table 4 presents the results of the base case of this evaluation. Considering a short-term time period, the average cost due to the incorporation of levosimendan in the cardiac-surgical process is 14,792 euros while the average cost per patient without levosimendan is 17,007 euros. The average cost saving difference per patient is 2275 euros.

**Table 4:** Base case results

	Cost per patient (€)	Mean ICU stay per patient (days)	Mean PO ward stay per patient (days)	Patients with no complications (% of total)
tervention (Levosimendan)	14,792.33	2.50	6.46	53.85
No intervention	17,006.94	5.70	6.40	31.71
Difference	2274.61	−3.20	0.06	22.14

ICU: Intensive Care Unit; €:euro; PO: post-operative

The average ICU stay per patient were estimated at 2.5 and 5.7 days for the levosimendan strategy and the absence of preconditioning respectively. Average stay in the postoperative ward per patient was estimated for the levosimendan strategy at 6.5 days and 6.4 days for the control group. Patients with no complications, any of them, for the levosimendan regimen were estimated at 53.8% of the total, while for the non-levosimendan regimen the estimate was 31.7% of the total.

### Deterministic sensitivity analysis

In this sensitivity analysis, the variable incidence of LCO depending on the definitions applied to the model. The cost per patient in the base case of 14,401 euros in the levosimendan group, compared to 16,652 euros in the control group, varies from 12,757 to 14,562 euros against 15,825 to 16,511 euros for a more restrictive definition of LCO, between 6 and 18% in the intervention group versus 25–35% in the control group. Similar tendencies are observed in ICU stay and hospital LOS, as well as percentage of complications presented, see Table 5.

**Table 5:** Deterministic sensitivity and probabilistic analysis

Deterministic sensitivity analysis	LCO incidence	Cost per patient (€)	Mean ICU stay per patient (days)	Mean PO ward stay per patient (days)	Patients with no complications (% of total)
Intervention (Levosimendan)	**Base case**	14,792.33	2.50	6.46	53.85
	LCO 18% [4]	14,562 [13,741-14,879]	2.35 [1.8–3]	6.22 [5.7–7.8]	55.01 [52.87–57.87]
	LCO 6% [17]	12,757 [12,172-14,892]	2.07 [1.4–2.3]	5.63 [4.9–7.5]	61.23 [55.69–68.71]
No intervention	**Base case**	17,006.94	5.70	6.40	31.71
	LCO 35% [16]	16,511 [15,061- 16,773]	5.3 [4.1–6.9]	6.1 [4.9–6.5]	35.22 [32.6–38.03]
	LCO 25% [4]	15,825 [14,988- 16,260]	4.8 [3.5.-5.7]	5.9 [4.8–6.3]	38.6 [35.7–41.32]
**Probabilistic analysis**	**Cost per patient (€)**		**Mean ICU stay (days)**	**Mean PO** **ward stay (days)**	**Patients with no complications (% of total)**
Intervention (Levosimendan)	14,401 [13,368-14,971]		2.39 [1–3.9]	6.23 [4.2–8.34]	51.8 [47.34–53.02]
No intervention	16,652 [16,004- 17,212]		5.4 [3.8–7.1]	6.17 [4.03–8.22]	29.98 [27.91–32.4]
Difference	2251				

€: euro, LCO: Low Cardiac Output, PO: postoperative, ICU: Intensive Care Unit, [95% CI]

[4] Ref. 4 (Mehta RH at al.) [16]. Ref 16 (Pieri M et al.) [17]. Ref 17 (Desai PM et al.)

### Probabilistic sensitivity analysis

Results of the probabilistic analysis are consistent with the estimates in the base case, resulting in savings or lower cost in the levosimendan group as a result of a lower number of postoperative complications. (Table 5).

**Fig. 2:**
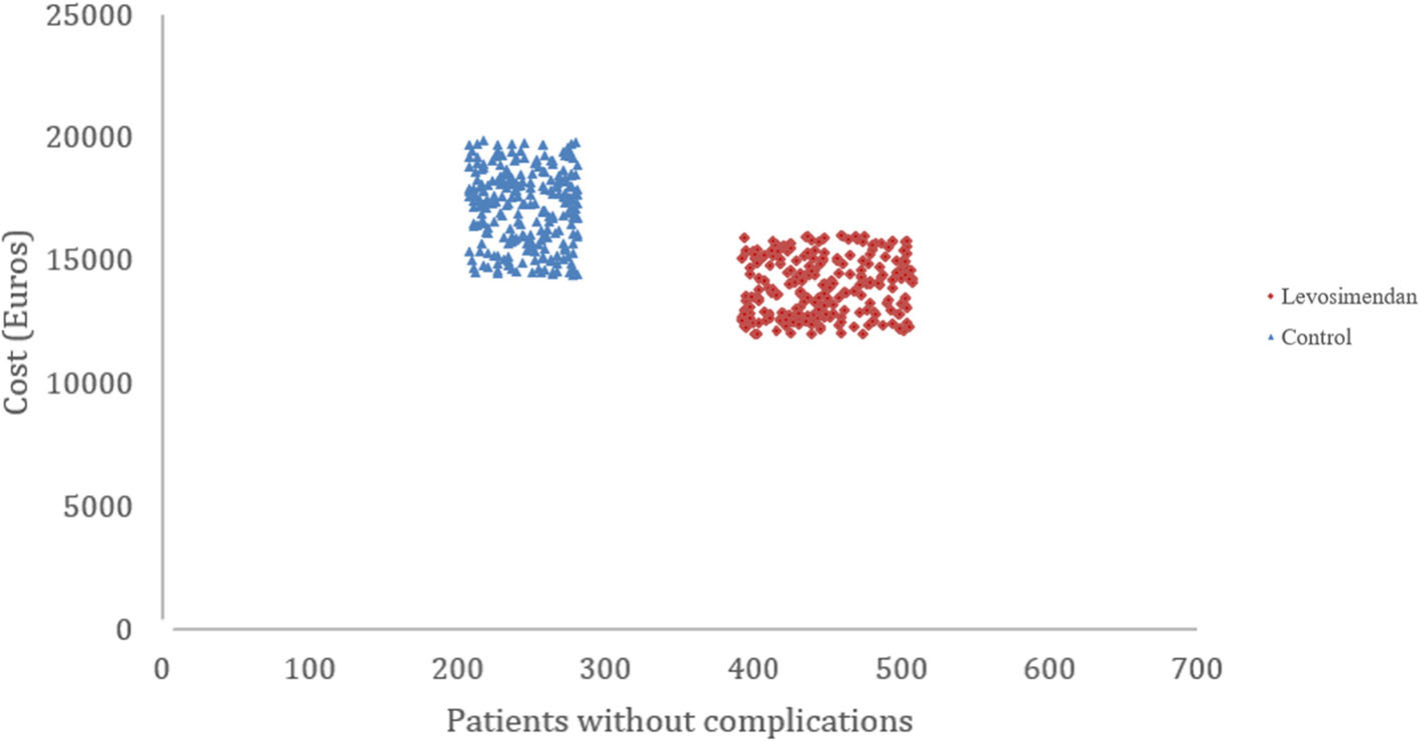
Cost-effectiveness plane

Figure 2 represents the cost and effectiveness plane displaying the cost and pairs obtained as a result of each of the Monte Carlo simulations. According to what was presented by the confidence intervals, we observed that in all the simulations, the use of preconditioning with levosimendan is less costly and more effective compared to control group.

## Discussion

The aim of this study is to demonstrate that the strategy of preconditioning with levosimendan in patients with moderate-severe systolic dysfunction is effective, not only reducing the appearance of postoperative low cardiac output but also being cost-effective, fundamentally reducing immediate postoperative stays in the ICU.For this reason, since this study is not a randomized study, we considered it necessary to exclude from the study patients who presented predictors of short term worse outcomes and prolonged postoperative ICU LOS. Among these, we highlight moderate-severe systolic dysfunction (LVEF < 40%), COPD, previous CABG, recent MI and urgency surgery and liver disease [18, 19].. Thus, we excluded patients presenting COPD, previous CABG and liver disease, as well as any type of urgent revascularization surgery derived from unstable angina or recent MI, to assess the short-term effect (low cardiac output) of preconditioning in patients with LVEF < 40% operated under an elective CABG surgery.

The data from our study are consistent with what is published in the literature regarding the efficacy of levosimendan use in impaired LVEF patients who will undergo CABG [4, 17, 20, 21], and shows that a preconditioning strategy with levosimendan decreases postoperative low cardiac output in patients with a compromised physiological myocardial reserve.

Administration of levosimendan in the perioperative period, has not demonstrated superiority in the improvement of survival, however it is still a useful drug for the treatment of LCO. Few studies have evaluated the preventive effect on the LCO in patients with moderate-severe systolic dysfunction [3, 4, 17, 21]. The beginning of the levosimendan infusion 48 h before the intervention, allows active metabolites to be effective in the span in which the onset of myocardial stunning is greater, meaning the first 24 h of the immediate postoperative phase [10]. This phenomenon is beneficial in preventing postoperative LCO; this effect can be observed for different stages of preoperative systolic dysfunction as evidenced in this study. In addition to the sustained hemodynamic effects, Levosimendan’s inhibition of intramitochondrial calcium accumulation associated with ischemia-reperfusion phenomena of extracorporeal circulation confers some myocardial protection [22, 23]. In this sense, levels of myocardial damage biomarkers, such as cTnI, have been described significantly lower in patients who have received preconditioning with Levosimendan [21], figures observed in our study, although they are not significant.

We observed lower lactate level at 24 h postoperatively in the Levosimendan group, similar to other studies [17], expression of better tissue perfusion in these patients. However, this drug exerts systemic vasodilator effect that sometime required vasopressor therapy to maintain an adequate perfusion pressure [21], which may condition a delay transfer from the ICU to the ward. On the other hand, the control group required a significant longer dosage of dobutamine, and norepinephrine. This group showed a higher significative incidence of cardiogenic shock. Several medical societies recommend the use of dobutamine and norepinephrine as first-line agents [24–26]. New advances in the physiopathology of cardiogenic shock show a decrease in peripheral vascular resistance through numerous pro-inflammatory pathways, thus leading to an indication of vasopressor therapy in these patients [27]. In addition, we must take into account that these pro-inflammatory pathways can be enhanced by extracorporeal circulation [28]. Norepinephrine increases mean arterial pressure without any concomitant increase in heart rate, and it also has a direct effect on cardiac myocytes as a result of beta 1 adrenergic receptor stimulation. Norepinephrine does not act on Beta2 adrenergic receptor, hence lactate levels do not increase and may be used to guide resuscitation [27]. In any case, our hemodynamic protocol establishes that inotropes and vasopressors should be applied at the lowest dose and shortest time span possible. Finally, levosimendan group showed less mechanical ventilation support, as similar studies [21].

As a consequence of the lower incidence of potential postoperative complications, preconditioned patients had a shorter ICU stay, following comparable results published by others authors [21, 29].

The economic evaluation carried out in this study, shows that levosimendan used as a preconditioning strategy is cost-effective in moderate-severe systolic dysfunction patients who will undergo elective CABG patients. This result is supported by both the lower cost associated with potential postoperative complications and a shorter LOS.

Several studies demonstrate the cost-effectiveness of levosimendan in patients with heart failure [30–33]. Nonetheless few studies have performed similar analysis in the cardiac surgery setting. An economic evaluation has recently been published in which the cost savings of levosimendan have been analysed against the use of dobutamine in the short and long term in patients undergoing cardiac surgery from the German national health care register [8]. In the mentioned study, medication and postoperative complications were the clinical variables studied and the cost-benefit in terms of costs and LOS were evaluated. The results showed a saving of 4787 euros per patient from the perspective of reduced postoperative complications and a shorter associated stay. This study, does not distinguish the stretch of time in which levosimendan was used, being a global perioperative analysis and the costs of complications were applied according to the related diagnostic group. The observed differences in cost-savings compared to the Marguidian study can be explained by several reasons. Our study focuses on the use of levosimendan as a preconditioning strategy and the reduction of potential postoperative CABG complications in a specific type of patient: those with moderate-severe systolic dysfunction without other known prolonged ICU stay risk predictors in CABG surgery [18], since they were excluded from the study. The increased in the complexity of these patients and, consequently, the risk of the appearance of adverse effects, triggered by LCO itself could, in turn, increase the benefit of this measure, since, for the calculation of costs, the observed frequency of mentioned adverse effects is of overriding importance. Preconditioning requires an earlier admission to a high-cost unit such as the coronary unit, which carries an added cost compared to the aforementioned study.

We have considered the benefit in global terms, i.e. reduction of complications and postoperative stay. These complications occur in the immediate postoperative period, which lead to prolonged ICU stay and increasing costs. To the above, we must take into account the limitation in the number of beds in the ICU, leading a more intensive use of the ICU with the potential problems that can result from this situation [34]. This aspect is of great interest since the use of levosimendan as a pre-conditioning strategy has been shown to reduce patient complications and therefore ICU LOS. This contributes to improving the turnover in a limited-bed high-level care unit, consequently, decrease number of surgical cancellations [35, 36], maximizing the overall cardiac surgical program throughputs when it is implemented.

### Limitations

The reduced sample size of patients receiving preconditioning may be one of the main limitations, we have been able to demonstrate the beneficial effect of preconditioning even when patients receiving levosimendan had a worse LVEF. This underlines the positive effect of this strategy in line with the results published in the literature to date.

In addition, the design of the sequential assignment study may introduce an unknown bias, so that we tried to reduce by excluding patients with well-known risk factors subjected to prolonged stays after surgery in the intensive care unit. There is no universal definition of postoperative LCO considered by all research groups, which may cause some variability in the incidence of complication above mentioned.

Costs include in the economic evaluation have been carried out at a local level, thus the transferability of results outside the context of the national health system could be compromised.

## Conclusions

Preconditioning with levosimendan in moderate to severe systolic dysfunction (LVEF < 40%), is associated with a lower incidence of LCO in the postoperative period of elective myocardial revascularization surgery, and this preventive procedure is cost-effective.

The efficiency of this strategy is determined by a lower consumption of resources in the immediate postoperative period which takes place in a high care level unit. A shorter stay in the ICU translates into a systemic benefit for the hospital environment, improving the flow and availability of high care level beds.

## Supplementary information

**Additional file 1.** Postcardiotomy Low Cardiac Output Management.

**Additional file 2.** Mechanical Ventilation Weaning And Extubation Procedures In Postcardiotomy Patients.

## Data Availability

The datasets used and/or analysed during the current study are available from the corresponding author on reasonable request.

## Abbreviations

CABG: Coronary Artery Bypass Graft
COPD: Chronic Obstructive Pulmonary Disease
cTnI: Cardiac Troponin I
ICU: Intensive Care Unit
LCO: Low Cardiac Output
LOS: Length of Stay
LVEF: Left Ventricular Ejection Fraction
MI: Myocardial Infarction
NOAF: New Onset Atrial Fibrillation

## References

[1] Windecker S, Kolh P, Alfonso F (2014). 2014 ESC/EACTS guidelines on myocardial revascularization: the task force on myocardial revascularization of the European Society of Cardiology (ESC) and the European Association for Cardio-Thoracic Surgery (EACTS) developed with the special contribution of the European Association of Percutaneous Cardiovascular Interventions (EAPCI). Eur Heart J.

[2] Velazquez EJ, Lee KL, Deja MA (2011). For the STICH investigators. Coronary-artery bypass surgery in patients with left ventricular dysfunction. N Engl J Med.

[3] Cholley B, Caruba T, Grosjean S (2017). Effect of Levosimendan on low cardiac output syndrome in patients with low ejection fraction undergoing coronary artery bypass grafting with cardiopulmonary bypass: the LICORN randomized clinical trial. J Am Med Assoc.

[4] Mehta RH, Leimberger JD, van Diepen S (2017). LEVO-CTS investigators. Levosimendan in patients with left ventricular dysfunction undergoing cardiac surgery. N Engl J Med.

[5] Landoni Giovanni, Lomivorotov Vladimir V., Alvaro Gabriele, Lobreglio Rosetta, Pisano Antonio, Guarracino Fabio, Calabrò Maria G., Grigoryev Evgeny V., Likhvantsev Valery V., Salgado-Filho Marcello F., Bianchi Alessandro, Pasyuga Vadim V., Baiocchi Massimo, Pappalardo Federico, Monaco Fabrizio, Boboshko Vladimir A., Abubakirov Marat N., Amantea Bruno, Lembo Rosalba, Brazzi Luca, Verniero Luigi, Bertini Pietro, Scandroglio Anna M., Bove Tiziana, Belletti Alessandro, Michienzi Maria G., Shukevich Dmitriy L., Zabelina Tatiana S., Bellomo Rinaldo, Zangrillo Alberto (2017). Levosimendan for Hemodynamic Support after Cardiac Surgery. New England Journal of Medicine.

[6] Altenberger J, Parissis JT, Costard-Jaeckle A (2014). Efficacy and safety of the pulsed infusions of levosimendan in outpatients with advanced heart failure (LevoRep) study: a multicentre randomized trial. Eur J Heart Fail.

[7] Gallego-Delgado M, Villacorta E, Valenzuela-Vicente MC (2019). Start-up of a Cardiology Day Hospital: Activity, Quality Care and Cost-effectiveness Analysis of the First Year of Operation. Rev Esp Cardiol (Eng Ed).

[8] Mardiguian S, Kivikko M, Heringlake M (2016). Cost-benefits of incorporating levosimendan into cardiac surgery practice: German base case. J Med Econ.

[9] Ávalos R., MartinezSanz R., Jiménez J. *et al.* Levosimendan preconditioning in patients undergoing elective cardiac surgery with poor ejection fraction. preliminary results. J Cardiothorac Surg. 2015;10:A310. 10.1186/1749-8090-10-S1-A310.

[10] Kivikko M, Lehtonen L, Colucci WS (2003). Sustained hemodynamic effects of intravenous levosimendan. Circulation.

[11] Lahtinen P, Pitkänen O, Pölönen P, Turpeinen A, Kiviniemi V, Uusaro A (2011). Levosimendan reduces heart failure after cardiac surgery: a prospective, randomized, placebo-controlled trial. Crit Care Med.

[12] Iribarren JL, Jiménez JJ, Barragán A (2009). Left atrial dysfunction and new-onset atrial fibrillation after cardiac surgery. Rev Esp Cardiol.

[13] Pérez Vela JL, Martín Benítez JC, Carrasco González M (2012). Clinical practice guide for the management of low cardiac output syndrome in the postoperative period of heart surgery. Med Int.

[14] Mehta RL, Kellum JA, Shah SV (2007). Acute kidney injury network. Acute Kidney Injury Network: report of an initiative to improve outcomes in acute kidney injury. Critical Care.

[15] Pulido JN (2017). Prediction of prolonged mechanical ventilation after cardiac surgery: an imperfect crystal ball. J Thorac Cardiovasc Surg.

[16] Pieri M, Belletti A, Monaco F (2016). Outcome of cardiac surgery in patients with low preoperative ejection fraction. BMC Anesthesiol.

[17] Desai PM, Sarkar MS, Umbarkar SR (2018). Prophylactic preoperative levosimendan for off-pump coronary artery bypass grafting in patients with left ventricular dysfunction: single-centered randomized prospective study. Ann Card Anaesth.

[18] Herman C, Karolak W, Yip AM, Buth KJ, Hassan A, Légaré JF (2009). Predicting prolonged intensive care unit length of stay in patients undergoing coronary artery bypass surgery--development of an entirely preoperative scorecard. Interact Cardiovasc Thorac Surg.

[19] Jacob KA, Hjortnaes J, Kranenburg G, de Heer F, Kluin J (2015). Mortality after cardiac surgery in patients with liver cirrhosis classified by the child-Pugh score. Interact Cardiovasc Thorac Surg.

[20] Sanfilippo F, Knight JB, Scolletta S (2017). Levosimendan for patients with severely reduced left ventricular systolic function and/or low cardiac output syndrome undergoing cardiac surgery: a systematic review and meta-analysis. Crit Care.

[21] Tritapepe L, De Santis V, Vitale D (2009). Levosimendan pre-treatment improves outcomes in patients undergoing coronary artery bypass graft surgery. Br J Anaesth.

[22] Vroom MB, van Wezel HB (1996). Myocardial stunning, hibernation, and ischemic preconditioning. J Cardiothorac Vasc Anesth.

[23] Sommer S, Leistner M, Aleksic I (2016). Impact of levosimendan and ischaemia-reperfusion injury on myocardial subsarcolemmal mitochondrial respiratory chain, mitochondrial membrane potential, Ca2+ cycling and ATP synthesis. Eur J Cardiothorac Surg.

[24] Levy B, Bastien O, Karim B (2015). Experts' recommendations for the management of adult patients with cardiogenic shock. Ann Intensive Care.

[25] Werdan K, Ruß M, Buerke M (2012). Cardiogenic shock due to myocardial infarction: diagnosis, monitoring and treatment: a German-Austrian S3 guideline. Dtsch Arztebl Int.

[26] Møller MH, Claudius C, Junttila E (2016). Scandinavian SSAI clinical practice guideline on choice of first-line vasopressor for patients with acute circulatory failure. Acta Anaesthesiol Scand.

[27] Levy B, Buzon J, Kimmoun A (2019). Inotropes and vasopressors use in cardiogenic shock: when, which and how much?. Curr Opin Crit Care.

[28] Jimenez JJ, Iribarren JL, Lorente L (2007). Tranexamic acid attenuates inflammatory response in cardiopulmonary bypass surgery through blockade of fibrinolysis: a case control study followed by a randomized double-blind controlled trial. Crit Care.

[29] Wang Benji, He Xiaojie, Gong Yuqiang, Cheng Bihuan (2018). Levosimendan in Patients with Left Ventricular Dysfunction Undergoing Cardiac Surgery: An Update Meta-Analysis and Trial Sequential Analysis. BioMed Research International.

[30] Lucioni C, D'Ambrosi A, Mazzi S (2012). Economic evaluation of levosimendan versus dobutamine for the treatment of acute heart failure in Italy. Adv Ther.

[31] Fedele F, D'Ambrosi A, Bruno N (2011). Cost-effectiveness of levosimendan in patients with acute heart failure. J Cardiovasc Pharmacol.

[32] de Lissovoy G, Fraeman K, Teerlink JR (2010). Hospital costs for treatment of acute heart failure: economic analysis of the REVIVE II study. The European Journal of Health Economics: HEPAC: Health Economics in Prevention and Care.

[33] Cleland JG, Takala A, Apajasalo M (2003). Intravenous levosimendan treatment is cost-effective compared with dobutamine in severe low-output heart failure: an analysis based on the international LIDO trial. Eur J Heart Fail.

[34] Green Linda V. (2002). How Many Hospital Beds?. INQUIRY: The Journal of Health Care Organization, Provision, and Financing.

[35] Arabi Y, Venkatesh S, Haddad S, Al Shimemeri A, Al MS (2002). A prospective study of prolonged stay in the intensive care unit: predictors and impact on resource utilization. Int J Qual Health Care.

[36] Almashrafi A, Elmontsri M, Aylin P (2016). Systematic review of factors influencing length of stay in ICU after adult cardiac surgery. BMC Health Serv Res.

