# Supplementary material for: Preconditioning with levosimendan reduces postoperative low cardiac output in moderate-severe systolic dysfunction patients who will undergo elective coronary artery bypass graft surgery: a cost-effective strategy
Source: J Cardiothorac Surg. 2020 May 24;15:108. doi: 10.1186/s13019-020-01140-z (PMC7245898; doi:10.1186/s13019-020-01140-z)

POSTCARDIOTOMY LOW CARDIAC OUTPUT MANAGEMENT

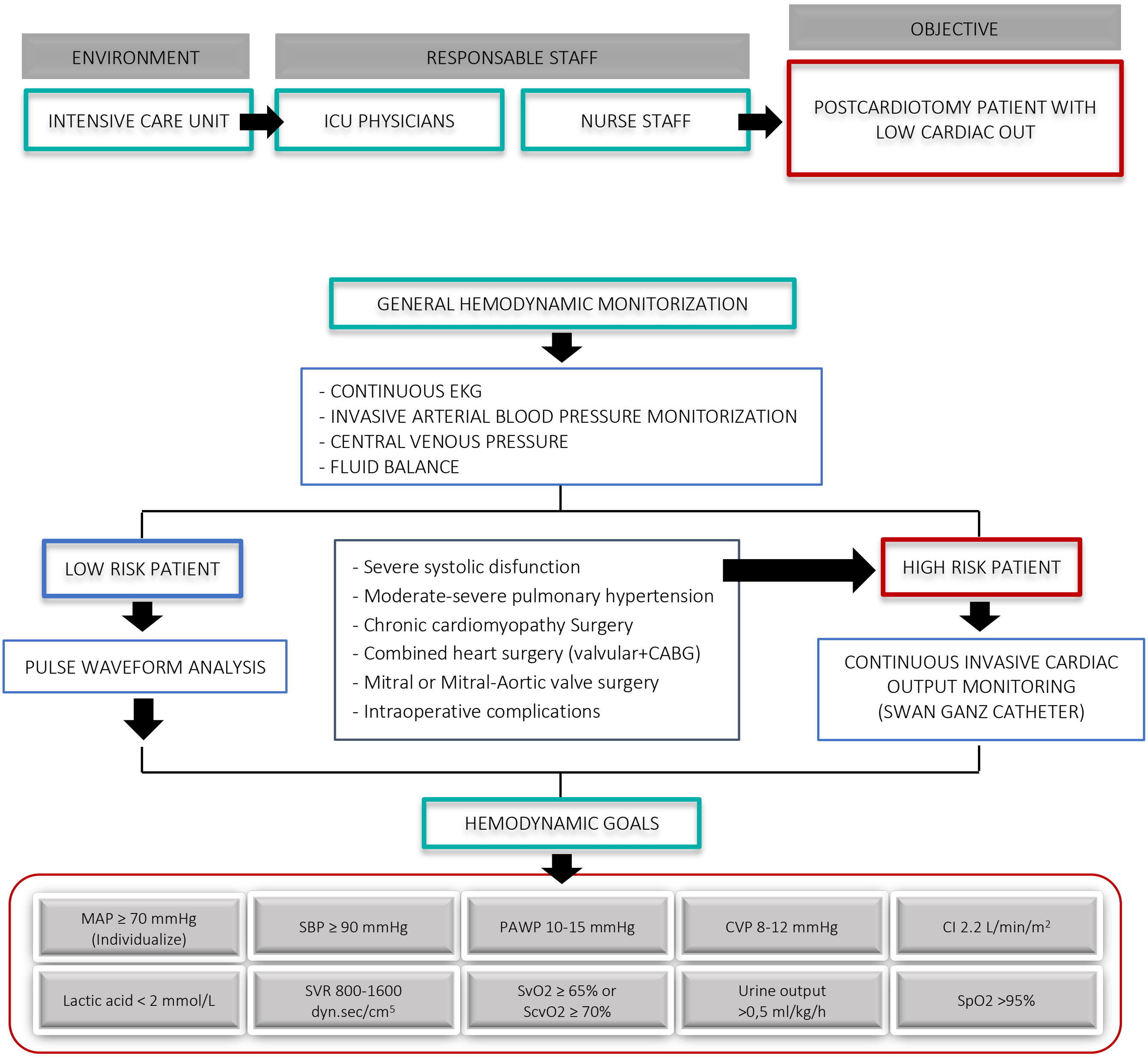

CABG: Coronary Artery Bypass Grafting; CI: Cardiac Index; CVP: Central Venous Pressure; EKG: Electrocardiogram; MAP: Mean Arterial Pressure; PAWP: Pulmonary Arterial Wedge Pressure; SBP: Systolic Blood Pressure; SvO2: Mixed Venous Oxygen Saturation; ScvO2: Central Venous Oxygen Saturation; SpO2: Peripheral Capillary Oxygen Saturation; SVR: Systemic Vascular Resistance.

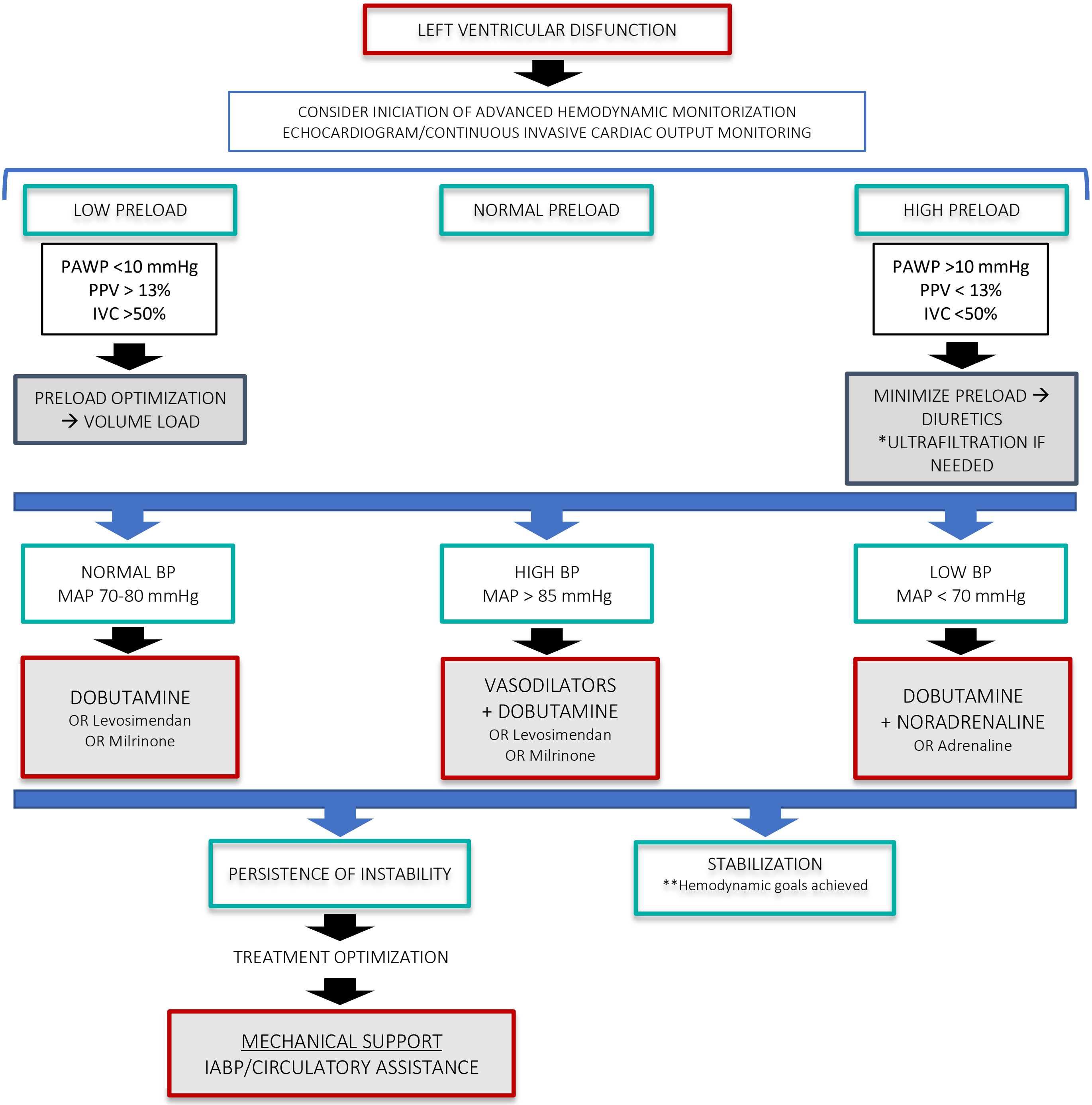

* Usually only one inotropic agent is used, but in selected cases, can be necessary the administration of more than one inotropic agent, seeking synergic effect.
** Hemodynamic goals were presented in the previous chart.

BP: Blood Pressure; IABP: Intra-aortic Balloon Pump; IVC: Inferior Vena Cava; MAP: Mean Arterial Pressure; PAWP: Pulmonary Arterial Wedge Pressure; PPV: Pulse Pressure Variation.

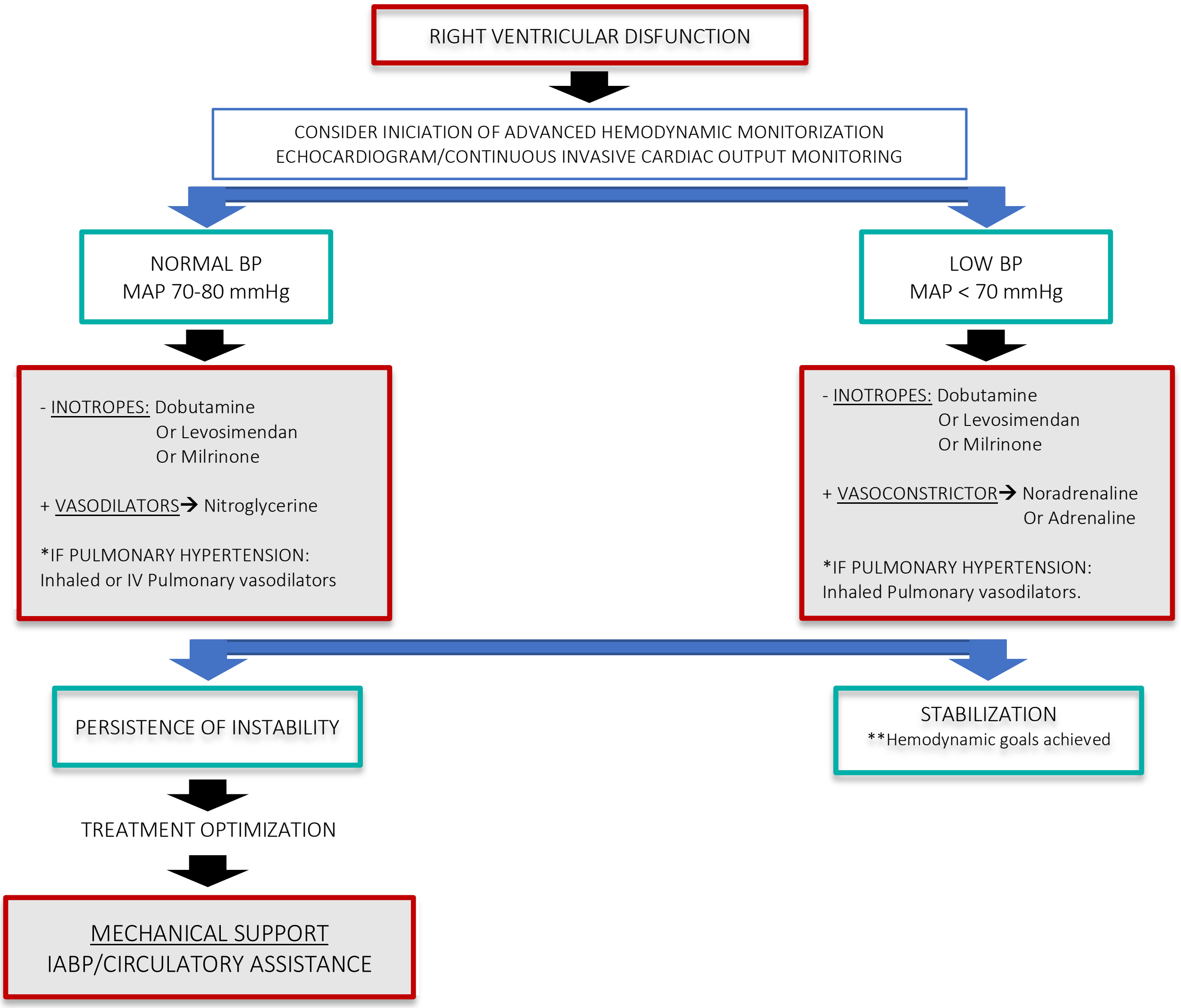

* If right ventricular filling volume is low or PAWP/CVP>1 and CVP <10 mmHg→ proceed to give volume load. Preload optimization with close monitorization and reevaluate. If PAWP/CVP <1 and rapidly increasing, or CVP >15 without cardiac output increase → DO NOT GIVE VOLUME LOAD.
** Hemodynamic goals were presented in the previous chart.

BP: Blood Pressure; CVP: Central Venous Pressure; IABP: Intra-aortic Balloon Pump; IV: Intravenous; MAP: Mean Arterial Pressure; PAWP: Pulmonary Arterial Wedge Pressure.

Servicio Canario de la Salud
HOSPITAL UNIVERSITARIO DE CANARIAS

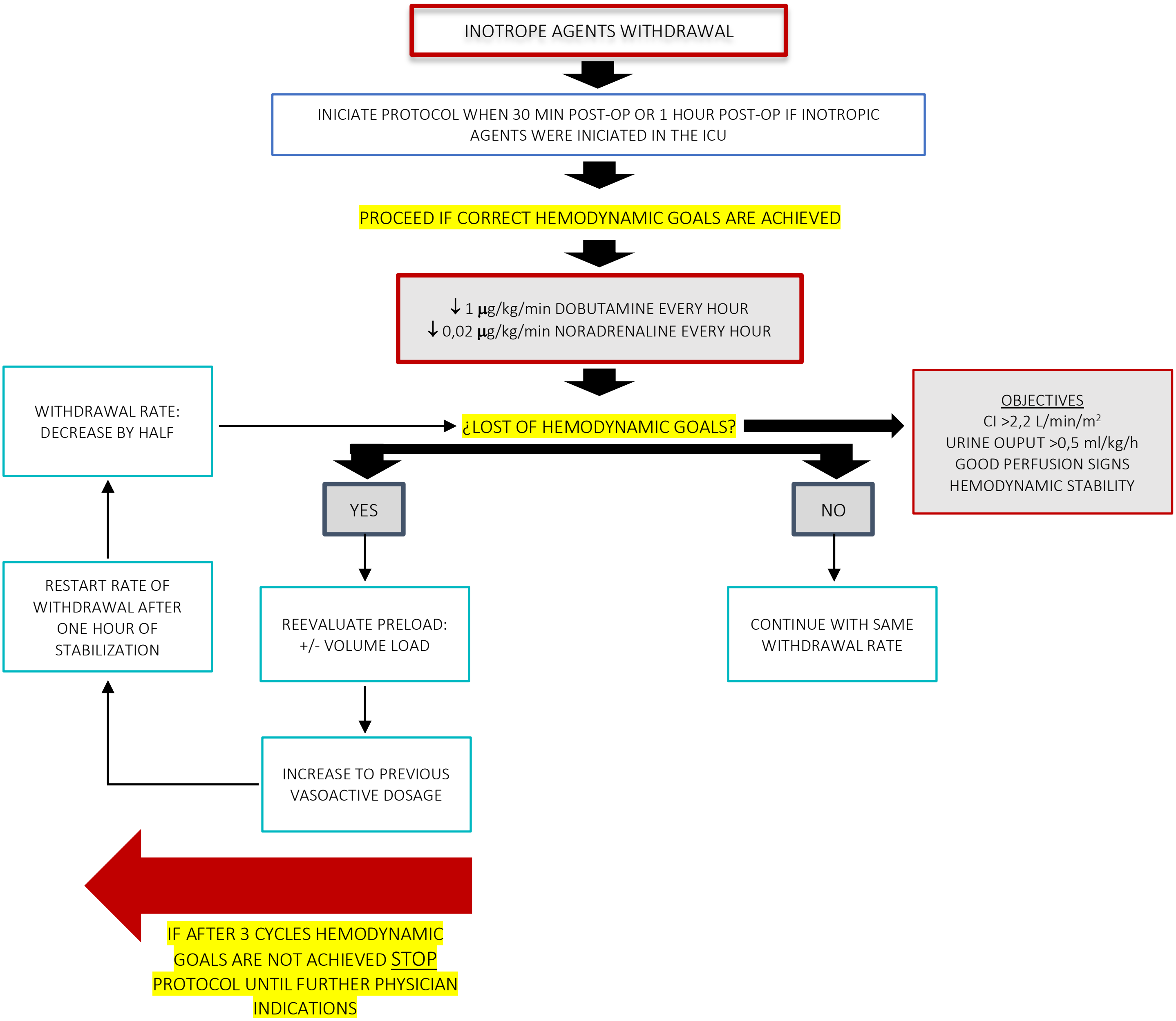

Supplement: Supplementary file 1 — Additional file 1. Postcardiotomy Low Cardiac Output Management. [file 13019_2020_1140_MOESM1_ESM.pdf]
