# Supplementary figures and images for: Preconditioning with levosimendan reduces postoperative low cardiac output in moderate-severe systolic dysfunction patients who will undergo elective coronary artery bypass graft surgery: a cost-effective strategy
Source: J Cardiothorac Surg. 2020 May 24;15:108. doi: 10.1186/s13019-020-01140-z (PMC7245898; doi:10.1186/s13019-020-01140-z)

## MECHANICAL VENTILATION WEANING AND EXTUBATION PROCEDURES IN POSTCARDIOTOMY PATIENTS

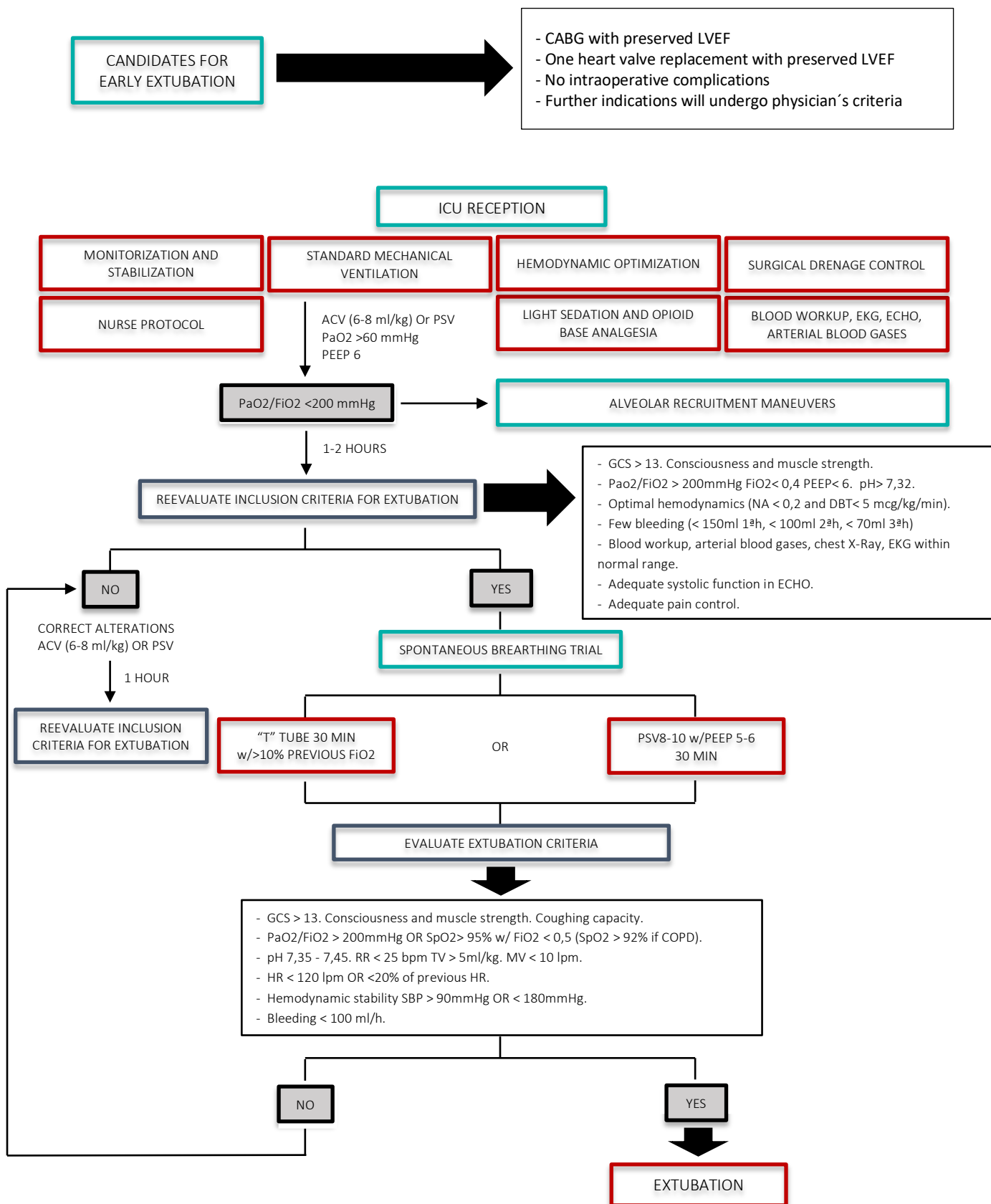

Supplement: Supplementary file 2 — Additional file 2. Mechanical Ventilation Weaning And Extubation Procedures In Postcardiotomy Patients. [file 13019_2020_1140_MOESM2_ESM.pdf]
